# Structural Properties and Macrophage Activation of Cell Wall Polysaccharides from the Fruiting Bodies of *Hericium erinaceus*

**DOI:** 10.3390/polym10080850

**Published:** 2018-08-01

**Authors:** Di Wu, Shan Yang, Chuan Tang, Yanfang Liu, Qiaozhen Li, Henan Zhang, Fengjie Cui, Yan Yang

**Affiliations:** 1 National Engineering Research Center of Edible Fungi, Key Laboratory of Edible Fungi Resources and Utilization (South), Institute of Edible Fungi, Shanghai Academy of Agriculture Sciences, Ministry of Agriculture, Shanghai 201403, China; wudi@saas.sh.cn (D.W.); yangshan624@126.com (S.Y.); tangchuan1990jy@163.com (C.T.); aliu-1980@163.com (Y.L.); liqiaozhen-345@163.com (Q.L.); henanhaoyun@126.com (H.Z.); 2 College of Food Science &Engineering, Shanghai Ocean University, Shanghai 201306, China; 3 School of Food and Biological Engineering, Jiangsu University, Zhenjiang 212013, China

**Keywords:** *Hericium erinaceus*, cell wall polysaccharides, structural properties, growth stages, immune activity in vitro

## Abstract

In this study, water-soluble and alkali-soluble cell wall polysaccharides were obtained from fruiting body extracted residual micropowders of *Hericium erinaceus*, harvested at seven different growing stages. The structural properties and in vitro immunity activities of cell wall polysaccharides extracted successively by hot water and sodium hydroxide solution were studied, and the results indicated that the yield and content of polysaccharides increased during the reproductive growth stage and decreased with the maturity of the fruiting body. Water-soluble cell wall polysaccharides mainly composed of glucose and galactose at a molar ratio of 3.4–14:1.0, and also contained a small ratio of glucuronic acid. The alkali-soluble cell wall polysaccharides were glucans with lower molecular weight and higher macrophage activation activity in vitro than water-soluble ones. Our findings suggest that the growth stages (H4 and H5) are suitable for harvesting *H. erinaceus* fruiting bodies with higher cell wall polysaccharide yield and functional benefits.

## 1. Introduction

The famous edible and medicinal mushroom *Hericium erinaceus*, also known as the Lion’s Mane Mushroom or Hedgehog Mushroom, belongs to the class of *Agaricomycetes* under the phylum basidiomycota [1]. It contains the numerous bioactive compounds, including polysaccharides, glycoproteins, sterols, terpenes, phenols, etc. [2,3,4,5,6]. Polysaccharides and polysaccharide–protein or peptide complexes are major bioactive macromolecules of *H. erinaceus* showing notable anti-tumor, immunomodulating, anti-inflammatory, anti-aging, antioxidative, and hepatoprotective effects, amongst other health benefits [7,8,9,10,11].

Generally, polysaccharides are classified as intracellular, extracellular, and cell wall polysaccharides [12,13,14]. After the extraction of intracellular polysaccharides from the mushroom fruiting body by hot water or other solvents, the residues are usually discarded without any further utilization. However, these residues are rich in cell wall polysaccharides, which have been proved as water-soluble, alkali-soluble, and alkali-insoluble glucans with a β-1,3, β-1,6 linkage structure showing significant immuno-stimulating, antioxidative, and renoprotective activities [15]. The hot water and alkali extraction of β-glucans in fungi or plant cell walls usually results in variations of their molecular weights, conformations, and biological activities [16,17,18]. However, to date, very few investigations are available about the structural characterization of cell wall polysaccharides from the fruiting bodies of *H. erinaceus*.

It is well-known that the development of fruiting maturity in mushrooms directly affects the chemical structure, content, and activity of bioactive compounds [19]. For example, the maturity of *Pleurotus eryngii* fruiting body increased the carbohydrate and protein contents and the antiproliferative effect of polysaccharide–protein fractions on SGC-7901 cells in vitro [20]. The mycelia growth stage I of *Pleurotus cornucopiae* favored the hemagglutinin lectin PCL-M synthesis, and the maturity to stage II and III decreased the PCL-M production [21]. Our group previously also found that the maturation stages significantly affected intracellular polysaccharide and protein contents in *H. erinaceus* fruiting bodies, and structures of water-extracted polysaccharides at stages of IV (small fungal spine stage), V (mid-fungal spine stage), and VI (mature) had a significant difference in the molecular weight distribution and monosaccharide compositions [22]. Nevertheless, an identical structural intracellular polysaccharide HPB-3, with molecular weight of 1.5 × 10^4^ Da and a backbone structure of α-1/6-linked galactopyranosyl connected to an α-fucopyranose side chain at the O-2 position was obtained from the fruiting body of *H. erinaceus* at IV, V, and VI maturating stages, respectively [23]. Our previous results showed that intracellular polysaccharides with low molecular weight were similar while large molecular weight polysaccharides showed many changes during the maturing period of *H. erinaceus*. However, the change rules of structures and biological activities of water- and alkali-soluble cell wall polysaccharides at different growth stages of *H. erinaceus* fruiting bodies are still unknown, and need to be further elucidated. Hence, the present study will aim to reveal the variation in structural properties of the cell wall polysaccharides extracted from *H. erinaceus* fruiting bodies at seven developmental stages, and find the possible relationship of cell wall polysaccharides with fruiting body maturity stage.

## 2. Materials and Methods

### 2.1. Materials and Chemicals

*Hericium erinaceus* strain 0605 was originated from the Edible Fungi Culture Collection Center Branch of the Agricultural Culture Collection of China (ACCC, Shanghai, China), and maintained on potato dextrose agar (PDA) (Sinopharm Chemical Reagent Co., Ltd., Shanghai, China) slants at 4 °C with periodic transfer.

Dulbecco’s Modified Eagle’s medium (DMEM), RPMI 1640 medium, fetal bovine serum (FBS), and trypsin were obtained from Gibco (Grand Island, NY, USA); penicillin and streptomycin were obtained from Amersco (Solon, OH, USA); dextran and monosaccharide standards and bacterial lipopolysaccharide (LPS) were obtained from Sigma-Aldrich Chemical Co. (St. Louis, MO, USA). Other chemicals and solvents were of analytical grade and used without further purification.

### 2.2. Fruiting Body Cultivation and Selection

**Figure 1:**
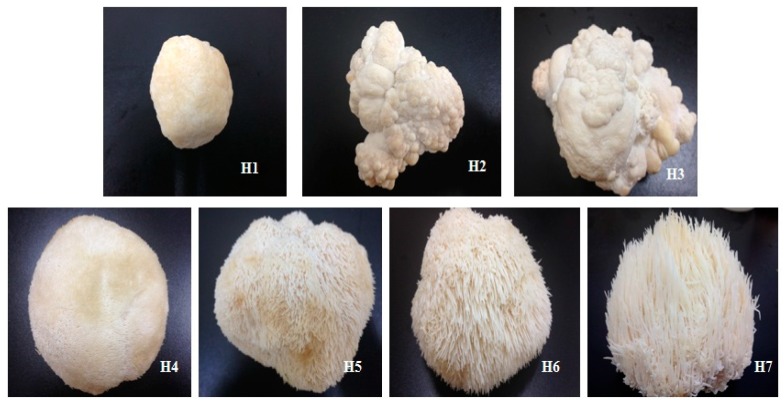
Morphologies of *Hericium erinaceus* fruiting bodies at seven growth stages (H1: bud-forming stage; H2: small lump stage; H3: split stage; H4: small fungal spine stage; H5: mid-fungal spine stage; H6: mature stage; H7: post-mature stage).

*H. erinaceus* was cultivated by Shanghai Guosen Biotechnology Co. Ltd. (Shanghai, China) with polypropylene bags containing the sterilized solid media (%, *w*/*w*): sawdust 30, corncob 16, cottonseed hull 22, wheat bran 15, corn starch 5, rice bran 10, and plaster 2. Mycelium was inoculated and kept in the dark at 25 °C and 90% relative humidity for 30 days, and laid on a ventilated field for 7 days. After germination, the fruiting bodies were harvested in seven stages of maturity: H1 (bud-forming stage), H2 (small lump stage), H3 (split stage), H4 (small fungal spine stage), H5 (mid-fungal spine stage), H6 (mature stage), and H7 (post-mature stage) (Figure 1).

### 2.3. Extraction of H. erinaceus Cell Wall Polysaccharides at Seven Developmental Stages

**Figure 2:**
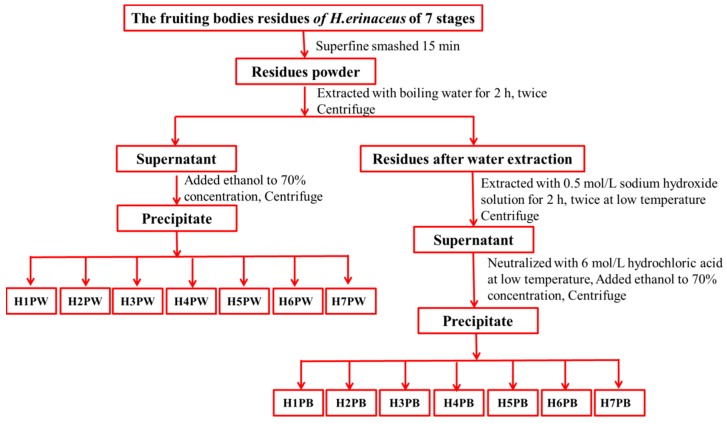
Stepwise extraction procedure of cell wall polysaccharides from *H. erinaceus* fruit bodies at 7 growth stages.

The cell wall polysaccharide was prepared from *H. erinaceus* fruiting bodies residues after water extraction at seven developmental stages, as shown in Figure 2. The air-dried residues (30 g) after extraction at seven stages were further ground for 15 min to break cell walls, and added to distilled water at a solid-to-solvent ratio of 1:20 and extracted at 100 °C for 2 h twice [24]. The supernatant was collected by centrifugation (15,317× *g*, 15 min), concentrated by evaporation under reduced pressure, and precipitated with ethanol to reach a final concentration of 70% at 4 °C for 12 h. Seven water-soluble cell wall polysaccharides were obtained after dialysis (3500 Da, 24 h) and freeze drying, and designated as H1PW, H2PW, H3PW, H4PW, H5PW, H6PW and H7PW. After hot water extraction, the residue powders (30 g) were further used for extraction with 0.5 mol/L sodium hydroxide solution with a solid-to-solvent ratio of 1:20 at 4 °C for 2 h twice. The supernatant was collected by centrifugation (15,317× *g*, 15 min), neutralized with 6 mol/L hydrochloric acid, concentrated by evaporation under reduced pressure, and precipitated with ethanol to reach a final concentration of 70% at 4 °C for 12 h. Seven alkali-soluble cell wall polysaccharides (H1PB, H2PB, H3PB, H4PB, H5PB, H6PB, and H7PB) were obtained after dialysis (3500 Da, 24 h) and were freeze dried and designated.

### 2.4. Physicochemical Properties and Structural Characterization of Cell Wall Polysaccharides

The total polysaccharides content was determined by phenol–sulfuric acid method [25] using D-glucose as a reference. Polysaccharide yield (%) was calculated by dividing the amount of extracted polysaccharide fraction by the weight of dried fruiting bodies.

The molecular weight of 14 polysaccharide fractions (seven water-soluble cell wall polysaccharides and seven alkali-soluble cell wall polysaccharides) was determined by high-performance size exclusion chromatography (HPSEC) (Waters, Milford, MA, USA). The system consisted of a Waters 2695 HPLC system equipped with multiple detectors: a refractive index detector (RI) and a UV detector for concentration determination, as well as a multiple-angle laser light scattering detector (MALLS, Wyatt Technology Co., Santa Barbara, CA, USA) for direct molecular determination. The columns were a TSK PWXL 6000 gel filtration column linked with a TSK PWXL 4000 gel filtration column, which were eluted with phosphate buffer (0.15 M NaNO_3_ and 0.05 M NaH_2_PO_4_, pH 7.0) at a flow rate of 0.5 mL/min. The calibration of the laser photometer was done with bovine serum albumin (BSA). A value of 0.146 mL/g was used as refractive index increment (d*n*/d*c*) for molecular weight calculation. Astra software (version 6.1.1, Wyatt Technology Co., Santa Barbara, CA, USA) was utilized for data acquisition and analysis. Column temperature and RI detector temperature were maintained at 35 ± 0.1 °C.

The monosaccharide composition of the 14 polysaccharide fractions and their ratios were determined using high-performance anion-exchange chromatography (HPAEC) with D-Gal, D-Glc, D-Ara, L-Fuc, L-Rha, D-Man, D-Xyl, D-Fru, D-Rib, D-GluA, and D-GalA (Sigma-Aldrich (St. Louis, MO, USA)) as the standards. Fourteen fractions (2 mg) were hydrolyzed with 4 mL 2 M trifluoroacetic acid (TFA) at 110 °C for 4 h. The monosaccharides were analyzed using a Dionex ICS2500 (Dionex, Sunnyvale, CA, USA) equipped with a CarboPacTM PA20 Analytical column (3 mm × 150 mm, Dionex, Sunnyvale, CA, USA). The column was eluted with 2 mM NaOH (0.45 mL/min) followed by 0.05 to 0.2 M NaAc at 30 °C.

### 2.5. Macrophage Activation

Macrophage activation was evaluated by NO production using the Griess method [26]. Mouse macrophages (RAW264.7), purchased from the American Type Culture Collection (ATCC, Manassas, VA, USA), were cultured in Dulbecco’s Modified Eagle’s medium (DMEM) containing 100 U/mL penicillin, 100 μg/mL streptomycin, and 10% fetal bovine serum (FBS) at 37 °C in a 5% CO_2_ humidified atmosphere. Aliquots (180 μL) of a RAW264.7 cell suspension (5 × 10^5^ cells/mL) were dispensed into each well of a 96-well plate together with 20 μL of the different test agents incubated at 37 °C for 48 h. Supernatants (100 μL) were reacted with 50 μL Griess reagent at room temperature for 10 min [22], and NO production was determined by measuring the absorbance at 543 nm using NaNO_2_ as the standard. The samples were previously dissolved in PBS at various concentrations (50, 200, and 500 μg/mL) before the tests. PBS and lipopolysaccharide (1 μg/mL) served as negative and positive controls, respectively.

### 2.6. Statistical Analysis

Each experiment was repeated three times using duplicate samples. The results were expressed as means ± standard deviations. Statistical comparisons were made by one-way analysis of variance (ANOVA), followed by Duncan’s multiple-comparison test. Differences were considered significant when the *p*-values were <0.05.

## 3. Results and Discussion

### 3.1. Yields and Contents of H. erinaceus Cell Wall Polysaccharides at Seven Developmental Stages

**Figure 3:**
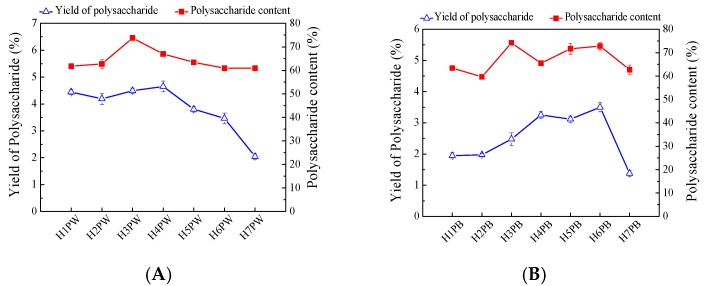
Yields and content of (**A**) water-soluble and (**B**) alkaline-soluble cell wall polysaccharides from *Hericium erinaceus* fruiting bodies at different growth stages.

The water- and alkali-soluble cell wall polysaccharides were extracted from the *H. erinaceus* residues at different developmental stages with hot water (100 °C) and alkali and precipitated by 70% ethanol, successively. As shown in Figure 3A, yields of the water-soluble cell wall polysaccharides significantly increased at the fourth stage, which reached 4.65%, and gradually decreased to 2.04% at stage H7. The maximum water-soluble cell wall polysaccharide content of 73.74% appeared at stage H3, with those of other stages being approximately 60% (Figure 3A).

Yields of alkaline-soluble cell wall polysaccharide showed an increasing trend from H1 to H6, and had the highest yield of 3.50% at the mature stage (H6) and then decreased to 1.38% at the post-mature stage, H7 (Figure 3B). The alkaline-soluble cell wall polysaccharide content reached the highest level of 74.19% at stage H3, maintained levels of approximately 70% at the mature stage, and then decreased to 62.69% at post-mature stages. These phenomena indicate that the early stages of *H. erinaceus* were mainly involved in the cell wall synthesis, mycelia growth, and development, while the late stages required carbohydrate catalysis to meet the energy requirements for maintaining mushroom growth. Our previous study also showed that the intercellular polysaccharide content of *H. erinaceus* increased with the maturation stage development of fruiting bodies, and attained the highest content at the mature stage (H6) [22]. These potentially indicate that a portion of cell wall polysaccharide transformed translated into intercellular polysaccharide during the late fruiting body maturation process. Similarly, the maturation of fruiting bodies also increased the carbohydrate content in *Agaricus bisporus* from 38.3% to 48.95% while it decreased the carbohydrate contents in fruiting bodies of both *Lactarius deliciosus* (L.) Gray and *Lactarius piperatus* (L.) Pers from 7.33% to 2.96% and 9.35% to 4.29%, respectively [27,28].

### 3.2. Molecular Weight Distribution of Cell Wall Polysaccharides of H. erinaceus at Different Developmental Stages

**Figure 4:**
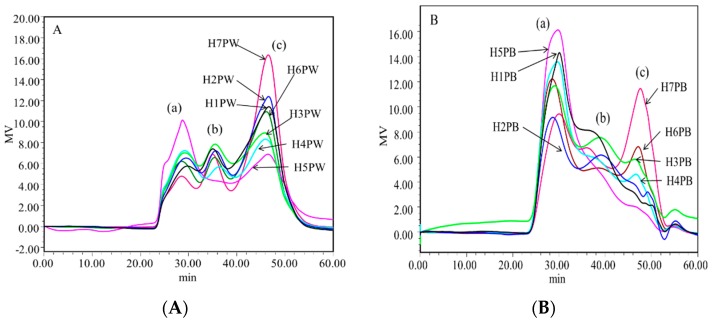
HPLC spectrogram of (**A**) water-soluble and (**B**) alkaline-soluble cell wall polysaccharide fractions from *Hericium erinaceus* fruiting bodies at different growth stages.

Molecular weight distributions of 14 polysaccharides of *H. erinaceus* are shown in Figure 4. The molecular weight and distribution trends of the cell wall polysaccharides at seven growth and development stages of *H. erinaceus* were similar, but the proportion of each fraction was significantly different. Each water-soluble (H1PW, H2PW, H3PW, H4PW, H5PW, H6PW, and H7PW) and alkali-soluble polysaccharide (H1PB, H2PB, H3PB, H4PB, H5PB, H6PB, and H7PB) was a polysaccharide mixture with three fraction peaks shown in HPLC chromatograms labeled as Peaks a, b, and c. By calculating with Astra data analysis software, the molecular weights and percentages of Peaks a, b, and c of 14 polysaccharides are summarized in Table 1. The molecular weight of peak a in water-soluble cell wall polysaccharides ranged from 8.77 × 10^6^ to 1.98 × 10^7^. The percentages of this fraction increased from 23.2% to 47.7% from stages H1 to H5, and then decreased to 19.2% in H7. The molecular weight of peaks b and c varied from 1.73 × 10^6^ to 3.85 × 10^6^, and from 3.17 × 10^5^ to 8.13 × 10^5^, respectively. The percentages of peak b in water-soluble cell wall polysaccharides were generally lower than those of peaks a and c. Table 1 also shows that the percentages of cell wall polysaccharides with lower molecular weight (peaks b and c) had higher levels in the early growth stages (H1 and H2) and later stages (H6 and H7), while having lower levels in maturity stages H3–H5. This indicates that the early growth stages had insufficient sources to synthesize the high-molecular-weight polysaccharides, while high-molecular-weight polysaccharides were synthesized as the energetic storage in the H4–H5 stages, then being broken down to a low-molecular-weight fraction for cell maintenance in the later stages.

The molecular weights of alkali-soluble polysaccharides (H1PB–H7PB) at seven stages were lower than those of water-soluble cell wall polysaccharides (Table 1). For example, the molecular weight of peak a in alkali-soluble polysaccharides ranged from 1.76 × 10^6^ to 2.76 × 10^6^, and those of peaks b and c varied from 3.43 × 10^5^ to 5.11 × 10^6^ and from 4.79 × 10^4^ to 3.52 × 10^5^, respectively. The percentages of peak a in alkali-soluble polysaccharides had the highest levels compared with those of peaks b and c. *H. erinaceus* fruiting bodies gave the maximum percentage (71.1%) of peak a and the minimum percentage (4.6%) of peak c at maturity stage H5. Similar trends were also observed in the late maturity stages (H6 and H7), where the percentages of peak a decreased to 40% while those of peaks b and c gradually increased to 29.8% and 29.7%, respectively, proving that *H. erinaceus* transferred to catabolic phases in the later stages of maturity.

### 3.3. Monosaccharide Compositions of Cell Wall Polysaccharides of H. erinaceus at Different Developmental Stages

Table 2 gives the monosaccharide composition changes of the cell wall polysaccharides of *H. erinaceus* at seven growth stages as determined by high-performance anion exchange chromatography (HPAEC). The water-soluble cell wall polysaccharides belonged to the heteropolysaccharides, containing glucose, galactose, fucose, and glucuronic acid. Among them, glucose and galactose were the main constituents, with molar ratios from 3.4 to 14. Fucose appeared mainly in the later maturity stages (H6 and H7) and glucuronic acid content rapidly increased to 109.10 μmol/g in that period.

Alkali-soluble cell wall polysaccharides were glucans with a sole composition of glucose at seven growth stages, which was consistent with the previous results showing that the alkali-soluble cell wall polysaccharide of mushroom sclerotium (*Pleurotus tuber-regium*) was a hyper-branched β-glucan [14]. Other results also proved that the polysaccharides present in the cell walls were mainly β-glucans in mushrooms [12,29]. However, the maturities of *H. erinaceus* fruiting bodies did not display clear indications as to the nature of changes in glucose content, varying from 1625.06 to 2080.93 μmol/g in stages H1–H7.

### 3.4. Macrophage Activation Activity of Cell Wall Polysaccharides of H. erinaceus at Different Developmental Stages

**Figure 5:**
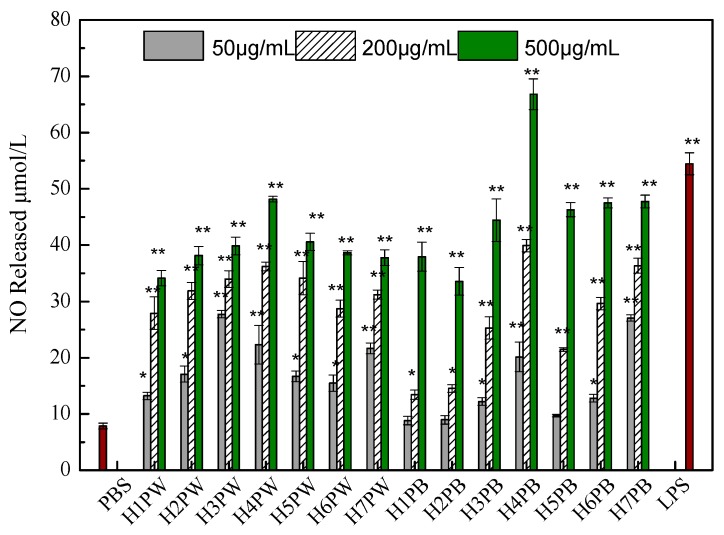
Effect of fruiting body maturity stage on NO production in murine macrophage-like cells RAW264.7. Cells (5 × 10^5^ cells/mL) were stimulated by cell wall polysaccharides. Supernatants were collected and NO production was determined using the Griess reagent. Each value represents the mean ± SD. * *p* < 0.05, ** *p* < 0.01 compared to the negative control (PBS treatment).

Generally, polysaccharides enhance and/or activate the immune responses of macrophage, leading to immunomodulation, anti-tumor activity, wound-healing and other therapeutic effects [30]. Herein, 14 cell wall polysaccharides extracted with hot water and alkali from *Hericium erinaceus* fruiting bodies growing at seven different stages of maturity were used to compare the macrophage immune activities by determining the NO production of RAW264.7 cells. As demonstrated in Figure 5, all polysaccharide fractions significantly stimulated the NO production of RAW264.7 cells in a dose-dependent manner. The water-soluble cell wall polysaccharide extracted from *H. erinaceus* at stage H4 showed the highest macrophage activation activity at the concentration of 500 μg/mL, while further increase of maturity stages (H5 to H7) only maintained a similar level of NO production. Similarly, the later maturity stages of *Agaricus brasiliensis* fruiting body seemed to be associated with decreases in its antioxidant activity [31] and anti-proliferation of Sarcoma 180 [32].

As for alkaline-soluble cell wall polysaccharides, low dose (50 μg/mL) and early growth stages (H1 and H2) could not favor their macrophage activation activities. With the increase of dose to 500 μg/mL and the maturity of *H. erinaceus* fruiting bodies (H4), NO productions were significantly activated to a maximum level (Figure 5). Our previous results also proved that the intracellular polysaccharide fraction H5FP6 isolated from the mature stage (H6) fruiting body had higher macrophage activation activity compared to those of other growth stages [22]. Hence, it could be proposed that the high-molecular-weight fractions and glucose content in polysaccharides (H4PW and H4PB) would benefit the macrophage stimulation and immunity-state activation.

## 4. Conclusions

Our study first reported on the changes in structural characteristics and macrophage activation of *H. erinaceus* cell wall polysaccharides with the development of the fruiting bodies’ maturity. The yield and content of water-soluble and alkali-soluble cell wall polysaccharides increased at the primary reproductive growth stage and then decreased in the later stages. The alkali-soluble cell wall polysaccharides were glucans with relatively lower molecular weight than those of water-soluble ones, and showed better macrophage activation activity at high dose (500 μg/mL). Hence, the growth stages (H4 and H5) are suitable for harvesting *H. erinaceus* fruiting bodies with high nutritional properties and market values. Further investigations should focus on finding the answers of how to synthesize *H. erinaceus* polysaccharides and how to realize the over-production of these polysaccharides.

## Figures and Tables

**Table 1:** Molecular weight distribution of water-soluble and alkaline-soluble cell wall polysaccharides from *H. erinaceus* fruiting bodies at different growth stages.

	Peak a			Peak b			Peak c		
	Mw (Da)	Mw/Mn	Ratio (%)	Mw (Da)	Mw/Mn	Ratio (%)	Mw (Da)	Mw/Mn	Ratio (%)
H1PW	1.19 × 10^7^	1.21	23.2	2.09 × 10^6^	1.20	25.1	3.17 × 10^5^	1.49	51.7
H2PW	8.77 × 10^6^	1.24	27.9	1.73 × 10^6^	1.10	24.1	3.18 × 10^5^	1.44	48.0
H3PW	1.07 × 10^7^	1.30	29.2	2.26 × 10^6^	1.22	30.4	4.12 × 10^5^	1.25	40.4
H4PW	1.08 × 10^7^	1.34	38.0	2.58 × 10^6^	1.11	22.8	6.14 × 10^5^	1.27	39.1
H5PW	1.28 × 10^7^	1.13	47.7	3.54 × 10^6^	1.08	17.9	8.13 × 10^5^	1.30	34.3
H6PW	1.45 × 10^7^	1.22	28.0	2.70 × 10^6^	1.12	23.3	5.11 × 10^5^	1.41	48.6
H7PW	1.98 × 10^7^	1.24	19.2	3.85 × 10^6^	1.16	23.4	4.53 × 10^5^	1.80	57.4
H1PB	2.10 × 10^6^	1.29	53.5	4.00 × 10^5^	1.28	39.0	1.02 × 10^5^	1.02	7.5
H2PB	2.74 × 10^6^	1.40	46.4	4.18 × 10^5^	1.31	42.0	1.15 × 10^5^	1.02	11.6
H3PB	2.41 × 10^6^	1.38	51.3	4.02 × 10^5^	1.19	37.3	1.42 × 10^5^	1.02	11.4
H4PB	2.46 × 10^6^	1.27	60.7	4.72 × 10^5^	1.22	28.4	1.05 × 10^5^	1.08	10.9
H5PB	2.11 × 10^6^	1.34	71.1	5.11 × 10^5^	1.02	24.2	3.52 × 10^5^	1.01	4.6
H6PB	2.37 × 10^6^	1.29	55.2	4.57 × 10^5^	1.13	27.7	1.37 × 10^5^	1.09	17.2
H7PB	1.76 × 10^6^	1.31	40.5	3.43 × 10^5^	1.18	29.8	4.79 × 10^4^	1.44	29.7

**Table 2:** Monosaccharide composition of water-soluble and alkaline-soluble cell wall polysaccharides from *Hericium erinaceus* fruiting bodies at different growth stages.

Sample	Fucose	Galactose	Glucose	Glucuronic Acid
H1PW	---	334.27	2182.25	92.96
H2PW	90.72	554.20	1886.96	78.09
H3PW	---	276.65	2093.30	80.32
H4PW	---	265.97	2195.48	78.25
H5PW	---	153.70	2126.49	43.85
H6PW	94.53	355.30	1917.93	109.10
H7PW	106.80	491.47	1914.19	102.82
H1PB	---	---	2080.93	---
H2PB	---	---	1625.06	---
H3PB	---	---	2030.18	---
H4PB	---	---	1826.03	---
H5PB	---	---	1937.56	---
H6PB	---	---	1778.14	---
H7PB	---	---	1996.60	---

Values are expressed as μmol/g polysaccharide; ---: not detected.
